# TMT-based proteomic analysis reveals integrins involved in the synergistic infection of reticuloendotheliosis virus and avian leukosis virus subgroup J

**DOI:** 10.1186/s12917-022-03207-6

**Published:** 2022-04-04

**Authors:** Xiyao Cui, Xinyue Zhang, Jingwen Xue, Yongxiu Yao, Defang Zhou, Ziqiang Cheng

**Affiliations:** 1 College of Veterinary Medicine, Shandong Agricultural University, Tai’an, 271018 China; 2 The Pirbright Institute & UK-China Centre of Excellence On Avian Disease Research, Pirbright, Ash Road, Guildford, GU24 0NF Surrey UK

**Keywords:** Reticuloendotheliosis virus, Avian leukosis virus subgroup J, TMT proteomic analysis, Synergistic infection

## Abstract

**Background:**

Co-infection with the avian leukosis virus subgroup J (ALV-J) and the reticuloendotheliosis virus (REV) increases mutual viral replication, causing a more serious pathogenic effect by accelerating the progression of neoplasia and extending the tumor spectrum. However, the molecular mechanism underlying the synergistic replication of ALV-J and REV remains unclear.

**Results:**

Here, we performed this study to compare the differentially expressed proteins among CEF cells infected with ALV-J, REV or both at the optimal synergistic infection time using TMT-based quantitative proteomics. We identified a total of 719 (292 upregulated and 427 downregulated) and 64 (35 upregulated and 29 downregulated) proteins by comparing co-infecting both viruses with monoinfecting ALV-J and REV, respectively. GO annotation and KEGG pathway analysis showed the differentially expressed proteins participated in virus-vector interaction, biological adhesion and immune response pathways in the synergistic actions of ALV-J and REV at the protein levels. Among the differentially expressed proteins, a large number of integrins were inhibited or increased in the co-infection group. Further, eight integrins, including ITGα1, ITGα3, ITGα5, ITGα6, ITGα8, ITGα9, ITGα11 and ITGβ3, were validated in CEF cells by qRT-PCR or western blot.

**Conclusions:**

These findings proved that integrins may be key regulators in the mechanism of synergistic infection of REV and ALV-J, which will provide more insight into the pathogenesis of synergism of REV and ALV-J at protein level.

**Supplementary Information:**

The online version contains supplementary material available at 10.1186/s12917-022-03207-6.

## Background

Synergism commonly occurs in nature when two or more unrelated oncogenic viruses infect the same host. In addition, numerous reports from clinical studies highlighted that retrovirus synergism occurs naturally in humans, cows, chicken and other vertebrates [1–5]. Notably, avian leukosis virus subgroup J (ALV-J) belongs to the genus *Alpharetrovirus* and family *Retroviridae*. The virus has been reported to spread in all species of chicken and is known to induce myelocytomas, hemangioma and fibrosarcoma [6–8]. On the other hand, reticuloendotheliosis virus (REV) belongs to the genus *Gammaretrovirus* and the family *Retroviridae.* The virus causes immunosuppression, the runting disease and lymphoma in a variety of avian hosts [9]. Moreover, co-infection with ALV-J and REV increases viral replication, causing a more serious pathogenic effect by accelerating the progression of neoplasia and extending the tumor spectrum [10–12]. Although the significance of co-infection with ALV-J and REV has attracted considerable attention, the synergistic mechanisms of these two viruses remain largely unclear.

Integrins are integral membrane proteins, and all alpha and beta subunits include a single transmembrane spanning helix [13]. Up to now, 18 α subunits and 9 β subunits have been identified, which form more than 20 integrins in different combinations. The cytoplasmic domains of the α and β subunits interact with a diversity of intracellular proteins, such as cytoskeletal proteins and kinases to promote signaling for tumor formation and metastasis [14–16]. In addition, conformational changes to integrin can elicit cell-signaling events that increase ligand affinity/avidity as well as tumor virus internalization and replication [17–19]. However, the association between integrins and the synergistic actions of REV and ALV-J has not been widely investigated.

Previous studies have identified synergistic infection of REV and ALV-J promotes virus replication in *vitro* [20]. The Illumina RNA deep sequencing indicates that the significantly differently expressed miRNAs participate in virus-vector interaction, energy metabolism and cell growth. Further comprehensive proteome analysis will provide more knowledge and deeper understanding of the synergistic mechanisms of ALV-J and REV. Consequently, we performed this study to compare the differentially expressed proteins among CEF cells infected with ALV-J, REV or both at the optimal synergistic infection time using TMT quantitative proteomics, which will provide more insight into the pathogenesis of synergism of REV and ALV-J at protein level.

## Results

### Protein profiling

**Fig. 1:**
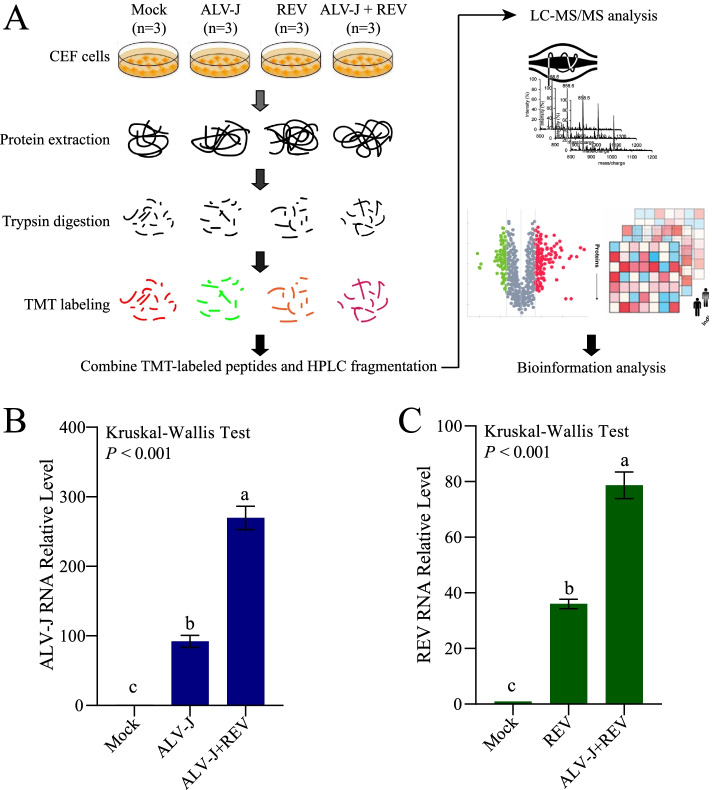
TMT-based quantitative proteomic analysis of the synergistic infection of ALV-J and REV.** A** Graphical illustration of the workflow used for TMT-based proteomic analysis. The same batch of samples were verified by qRT-PCR with ALV-J **B** or REV **C** specific primers

Our previous studies showed both ALV-J and REV levels in the co-infection group were increased significantly compared to those in the single infection groups at 48 hpi, 72 hpi, 96 hpi, 120 hpi and 144 hpi and reached the highest peak at 72 hpi [20].To further explore the synergistic mechanisms of REV and ALV-J, we also performed this study to compare the differentially expressed proteins among CEF cells infected with REV, ALV-J or both at 72 hpi using TMT quantitative proteomics (Fig. 1A). The same batch of samples were verified by qRT-PCR with ALV-J or REV specific primers (Fig. 1B and C). After processing MS/MS spectra in Maxquant software, 43,912 unique peptides were mapped to 6871 proteins in total, among which 4788 proteins were quantified (each group comprising 3 biological replicates). All the annotation and quantification information were presented in the Additional file 1: Table S1.

### Identification of differentially expressed proteins

**Fig. 2:**
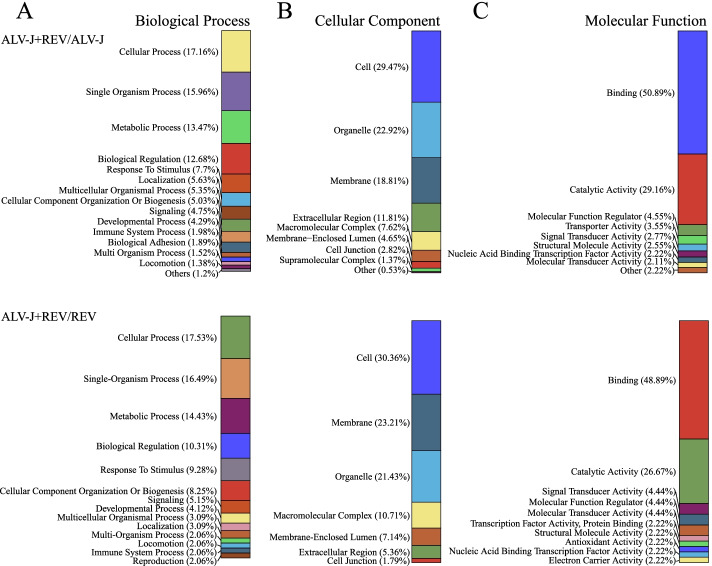
The different expressed proteins between co-infecting both viruses and monoinfecting ALV-J or REV. Volcano plot for proteins between co-infecting both viruses and monoinfecting ALV-J **A** and REV **B.** The proteins that are significantly changed (*p* < 0.05) are shown in the upper left corner (ratio < 0.677) and upper right corner (ratio > 1.5). **C** Heatmap of different expressed proteins for monoinfecting ALV-J vs Mock, monoinfecting REV vs Mock, and co-infecting both viruses vs Mock. **D** Venn diagrams of different expressed proteins

**Fig. 3:**
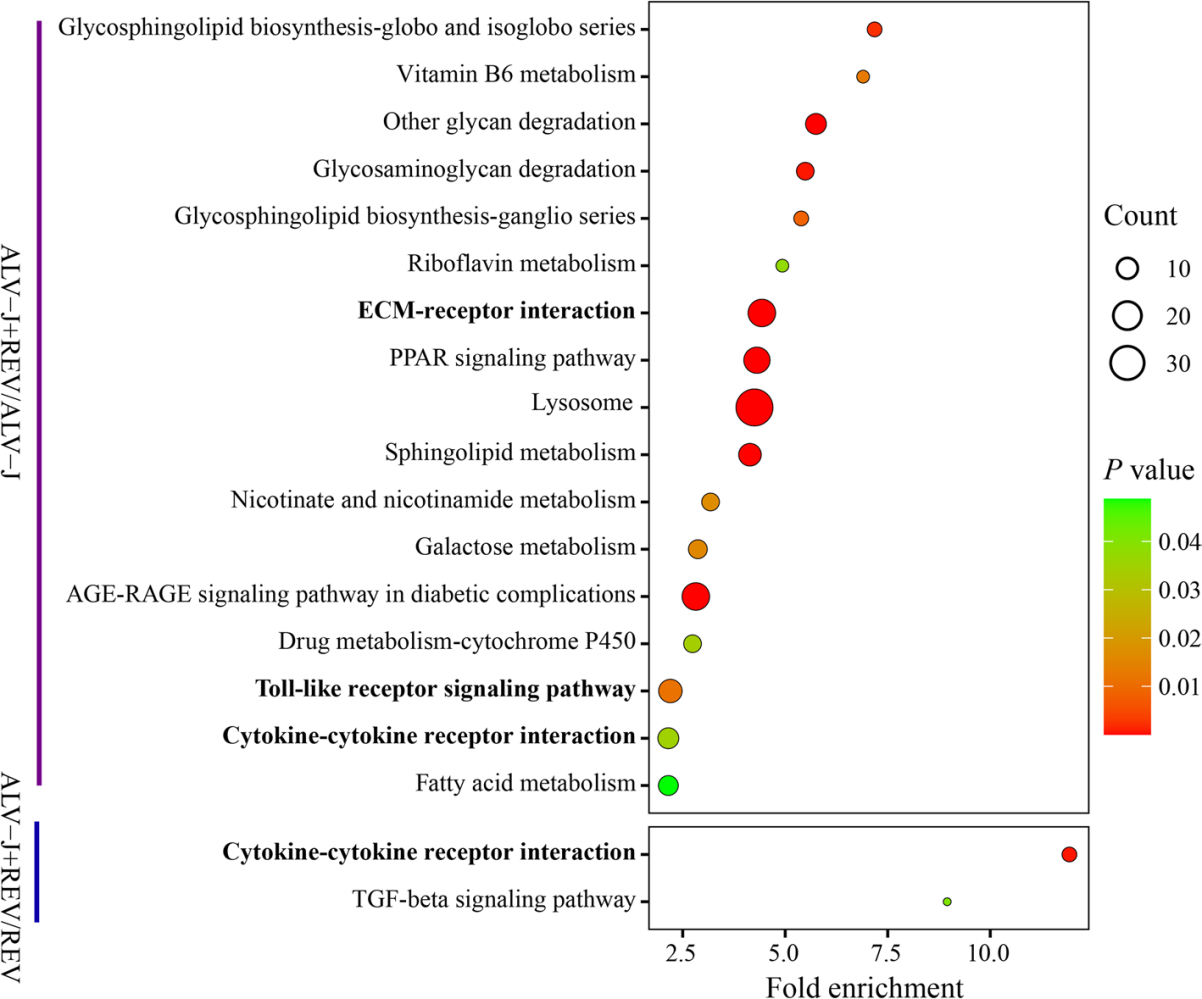
KEGG pathway analysis of different expressed proteins by comparing co-infection with two viruses with ALV-J-infected and REV-infected. Copyright permission of KEGG pathway maps were kindly provided by the Kanehisa laboratory in number 220181

Based on a cutoff of 1.2-fold change and *p* value < 0.05, a total of 719 (427 downregulated and 292 upregulated) and 64 (29 downregulated and 35 upregulated) proteins were identified by comparing co-infecting both viruses with monoinfecting ALV-J and REV, respectively (Fig. 2, Tables S2 and S3). These proteins were annotated by GO analysis to be involved in cellular (17% and 15%), single-organism (16% and 13%), and metabolic processes (13% and 11%) as well as biological regulation (13% and 12%, Fig. S1A). The proteins were also predicted to be components of cell structures (29% and 30%), organelles (23% and 20%), and macromolecular complexes (8% and 9%, Fig. S1B). Some proteins were molecular function regulator (4% and 6%) while others were involved in binding (51% and 52%), catalysis (29% and 24%), and signal transducer (3% and 5%, Fig. S1C). To further analyse the roles in regulatory networks, the different proteins were assigned to KEGG pathways utilizing the KEGG GENES Database [21–23]. The results implied that the most abundant KEGG terms were related to cytokine-cytokine receptor interaction, ECM-receptor interaction and Toll-like receptor signaling (Fig. 3). These findings proved that the differentially expressed proteins play important ruler roles in virus-vector interaction, biological adhesion and immune response.

### Integrins are associated with the synergistic infection of REV and ALV-J

**Fig. 4:**
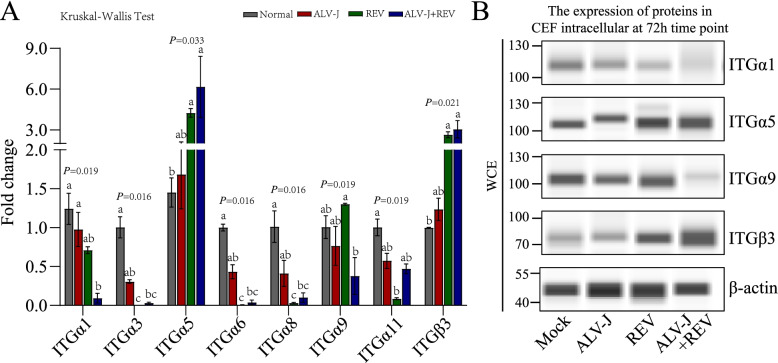
Integrins are associated with the synergistic infection of REV and ALV-J.** A** The qRT–PCR results of eight Integrins, including ITGα1, ITGα3, ITGα5, ITGα6, ITGα8, ITGα9, ITGα11 and ITGβ3 in CEFs, were consistent with the TMT-based proteomic analysis. The data represent the mean ± SEM determined from three independent experiments (*n* = 3), with each experiment containing three technical replicates. **B** ALV-J and REV synergistically enhanced the ITGα5 and ITGβ3 protein levels, and declined ITGα1 and ITGα9 protein levels in CEFs at 72 hpi as detected by western blot with anti-ITGα5 antibody, anti-ITGβ3 antibody, anti- ITGα1 antibody and anti-ITGα9 antibody. Western blot images obtained subsequent to running a Simple Western™ System (ProteinSimple)

Among the different expressed proteins between co-infecting both viruses and monoinfecting ALV-J or REV, integrins were upregulated or downregulated to various extents. As multifunctional heterodimeric cell-surface receptor molecules, integrins have been shown to usefully serve as entry receptors for a plethora of viruses. Previous studies showed Toll-like receptors increased expressions of integrins, which contributed to tumor formation through interaction of integrins with the ECM [24–27]. To make sure the results of proteomics, eight integrins, including ITGα1, ITGα3, ITGα5, ITGα6, ITGα8, ITGα9, ITGα11 and ITGβ3 that altered significantly in the co-infection group compared to each monoinfection group, were choose for qRT-PCR analysis with primers in Table 1. After RNA was isolated from CEF cells infected with REV, ALV-J and both at 72 hpi, all 8 integrins showed RNA expression profiles in CEFs that were in agreement with the TMT-based proteomic analysis (Fig. 4A). Further, Western blot verified REV and ALV-J synergistically increased proteins expression of ITGα5 and ITGβ3, declined protein expressions of ITGα1 and ITGα9 (Fig. 4B). These data verify that integrins are associated with the synergistic infection of REV and ALV-J.

**Table 1:** The primer sequences of Real-time PCR

Gene	Primer	Primer sequence	Size of PCR product
REV	F	TTGTTGAAGGCAAGCATCAG	105 bp
	R	GAGGATAGCATCTGCCCTTT	
ALV-J	F	TGCGTGCGTGGTTATTATTTC	144 bp
	R	AATGGTGAGGTCGCTGACTGT	
ITGα1	F	CCAGTAGGAAGAGACAGCCAAT	161 bp
	R	TAAGCATAGAGCGGTCCACAT	
ITGα3	F	CTCAACCTCACGCTGCTGGA	121 bp
	R	GCACTTCTGACTTCGCCTTCTT	
ITGα5	F	GTACATCTACAGCGGGAGGG	132 bp
	R	TTGCCATCCAGGTCGGTGT	
ITGα6	F	GGTTCCTGTCAGCAAGGTGTT	186 bp
	R	CTTATCTTGGCGGCTCTCATCA	
ITGα8	F	GTGGAAGGAGGAGCGGTGTA	110 bp
	R	GGTTCTCTGGTGCCATTGACTT	
ITGα9	F	GCAGGCTTCTTCACCGAGGA	195 bp
	R	ATCCGTGGTAGTTGGCTGAGAG	
ITGα11	F	CTTCGTCTGCTTCACTGCCATC	132 bp
	R	TGCCGCTCACCACTCTCATC	
ITGβ3	F	ACTTCTCCTGTGTCCGCTTCAA	101 bp
	R	GCAGTAGTCACCAGTCCAGTCT	
GADPH	F	GAACATCATCCCAGCGTCCA	132 bp
	R	CGGCAGGTCAGGTCAACAAC

## Discussions

Simultaneous infection by two retroviruses is not uncommon. Synergism of REV and ALV-J leads to accelerated neoplasia progression, and even extended tumor spectrum [28–30]. Our previous studies identified REV and ALV-J synergistically increase the accumulation of exosomal miRNAs [20]. It is well known that the biological functions of the miRNAs depend on the protein levels of the target genes, so studies at the proteome level may give a more realistic view of the synergistic mechanisms of ALV-J and REV. In the present study, we distinguished a total of 719 and 64 proteins by comparing co-infecting both viruses with monoinfecting ALV-J and REV, respectively. GO enrichment analysis demonstrated that most differentially expressed proteins took part in binding function. KEGG pathway analysis revealed that cytokine-cytokine receptor interaction, ECM-receptor interaction and Toll-like receptor signaling were the most abundant KEGG terms, meaning virus-vector interaction, biological adhesion and immune response may play significant roles in the synergistic actions of REV and ALV-J.

Both REV and ALV-J are classical oncogenic retroviruses, which co-infect the same flocks, same tissues and same cells, causing a more rapid neoplasia progression and extending tumor spectrum [10–12]. Previous Illumina RNA deep sequencing revealed the main differentially expressed miRNAs partook in energy metabolism, oxidative phosphorylation, virus-vector interaction and cell growth [20]. Our present study was in accord with that result, which indicated involvements of virus-vector interaction, biological adhesion and immune response pathways in the synergistic actions of ALV-J and REV at the protein levels. Though the exact mechanisms underlying accelerating neoplasia progression and extending the tumor spectrum are still unknown, virus-host interaction, cell adhesion and immunosuppression have been widely considered to be the key roles in tumor formation [31], indicating these differentially expressed proteins may also play a crucial role in the synergistic actions of REV and ALV-J.

The enhancement of viral transcription and protein expression was another characteristic of co-infection of REV and ALV-J. For viral replication, retroviruses integrate into the host genome to ensure viral persistence, which needs particular conditions for virus-vector interaction [32]. Thus, the roles of some host regulation factors that promote virus-host binding in co-infection of REV and ALV-J need to be explored. In present study, compared to monoinfection, TMT-based proteomic analysis showed a lot of integrins were inhibited or increased in the co-infection group, such as ITGα1, ITGα3, ITGα5, ITGα6, ITGα8, ITGα9, ITGα11 and ITGβ3, which has been demonstrated as an oncogene or a tumor suppressor gene in various tumors, respectively [33–41]. As multifunctional heterodimeric cell-surface receptor molecules, integrins have been shown to usefully serve as entry receptors for a plethora of viruses [42, 43], which means ALV-J and REV may synergistically regulate integrins for promoting viral replication. Therefore, the mechanism of integrins mediates ALV-J and REV synergistic infection needs to be further explored.

## Conclusions

In summary, a total of 719 and 64 proteins by comparing co-infecting both viruses with monoinfecting ALV-J and REV were identified by TMT quantitative proteomics, respectively, which participated in virus-vector interaction, biological adhesion and immune response pathways. Further, the abnormal expressions of ITGα1, ITGα3, ITGα5, ITGα6, ITGα8, ITGα9, ITGα11 and ITGβ3 were verified by qRT-PCR and western blot, indicating these integrins may be key regulators in tumor formation and metastasis processes induced by co-infection of REV and ALV-J. These findings will lead to further exploration of the mechanism of synergistic infection of REV and ALV-J.

## Methods

### Sample preparation

Chicken embryo fibroblasts (CEFs) cells were maintained in Dulbecco’s modified Eagle’s medium (DMEM) supplemented with 1% penicillin/streptomycin, 1% l-glutamine, 10% foetal bovine serum (FBS), and in a 5% CO_2_ incubator at 37℃. The stock SNV strain of REV at 10^3.2^ 50% tissue culture infectious doses (TCID_50_) and NX0101 strain of ALV-J at 10^3.8^ TCID_50_ were maintained in our laboratory. The TCID_50_ of the SNV and NX0101 strains were titrated by limiting dilution in DF-1 culture. The SPF chicks were purchased from Jinan SIPAFAS Poultry Co. Ltd. in Jinan, China. Cells (5 × 10^5^) of the same chicken embryo infected with ALV-J, REV or both (*n* = 3) were collected at 72 hpi, which was the optimal synergistic infection time [20]. Samples from uninfected 72 hpi were used as control. The same batch of samples were verified by qRT-PCR with ALV-J or REV specific primers (Table 1).

### TMT-labeled LC − MS/MS

The samples were sent to Hangzhou PTM Biolabs (Hangzhou, Zhejiang province, China) for TMT quantitative proteomics using the Maxquant search engine (v.1.5.2.8). In brief, each sample was sonicated three times on ice in lysis buffer (8 M urea, 1% Protease Inhibitor Cocktail). The remaining debris was removed and the protein concentration determined with a Pierce BCA protein assay kit (Thermo Fisher Scientific). After trypsin digestion, peptide was reconstituted in 0.5 M TEAB and processed according to the manufacturer’s protocol for TMT kit. LC–MS/MS data and bioinformatics analysis were performed as previously described [44, 45].

### Real-time quantitative reverse transcription polymerase chain reaction

Total RNA from CEF cells were isolated using the Tiangen RNeasy mini kit reverse and transcribed to cDNA using the TaqMan Gold Reverse Transcription kit as described in a previous study [20]. Real-time RT-PCR (qRT-PCR) was carried out using SYBR® Premix Ex TaqTM, and ITGα1, ITGα3, ITGα5, ITGα6, ITGα8, ITGα9, ITGα11, ITGβ3, ALV-J or REV specific primers (Table 1). All values were normalized to the endogenous control GAPDH to control for variation.

### Western blotting

ITGα1, ITGα5, ITGα9, and ITGβ3, were detected by simple western analysis [20] with anti-ITGα1 antibody, anti-ITGα5 (Bioss) antibody, anti-ITGα9 (Bioss) antibody, and anti- ITGβ3 (Bioss) antibody at a 1:1000, 1:1000, 1:1000 and 1:1000 dilution, respectively. Beta-actin was used as a loading control.

### Statistical analysis

Results are presented as the mean ± standard deviation(s). Statistical tests were performed using Non-parametric Kruskal–Wallis analysis using SPSS 13.0 statistical software. A P value less than 0.05 was considered statistically significant.

## Supplementary Information

**Additional file 1: Fig. S1.** Gene ontology analysis of 719 and 64 abnormal  expressed proteins by comparing co-infecting both viruses with monoinfecting ALV-J and REV, respectively. Proteins were annotated by biological Process, cellular Component and molecular Function. 

**Additional file 2: Fig. S2. **The original blots of Fig. 4B.

**Additional file 3: Table S1.** The significant differentially expressed proteins were quantified by analyzing the MS/MS spectra.

**Additional file 4: Table S2.** The significant differentially expressed proteins were identified by comparing co-infection with both viruses and infection with ALV-J only.

**Additional file 5: Table S3.** The significant differentially expressed proteins were identified by comparing co-infection with both viruses and infection with REV only.

## Data Availability

The mass spectrometry proteomics data have been deposited to the ProteomeXchange Consortium (http://proteomecentral.proteomexchange.org) via the iProX partner repository [46] with the dataset identifier PXD031503. ‍ The datasets used and analysed during the current study is available from the corresponding author on reasonable request.
