# Supplementary figures and images for: TMT-based proteomic analysis reveals integrins involved in the synergistic infection of reticuloendotheliosis virus and avian leukosis virus subgroup J
Source: BMC Vet Res. 2022 Apr 4;18:131. doi: 10.1186/s12917-022-03207-6 (PMC8978386; doi:10.1186/s12917-022-03207-6)

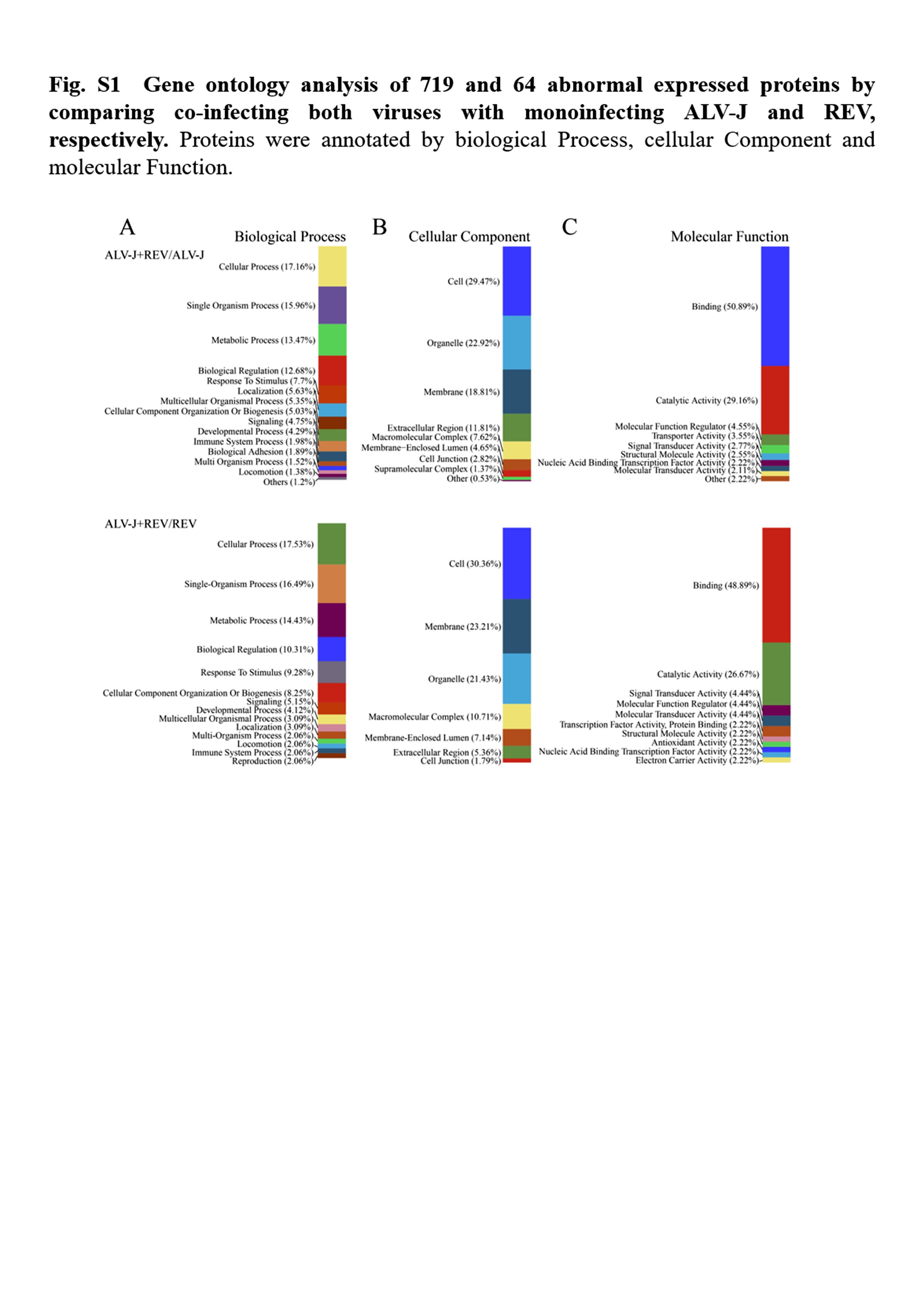

Supplement: Supplementary file 1 — Additional file 1: Fig. S1. Gene ontology analysis of 719 and 64 abnormal expressed proteins by comparing co-infecting both viruses with monoinfecting ALV-J and REV, respectively. Proteins were annotated by biological Process, cellular Component and molecular Function. [file 12917_2022_3207_MOESM1_ESM.png]

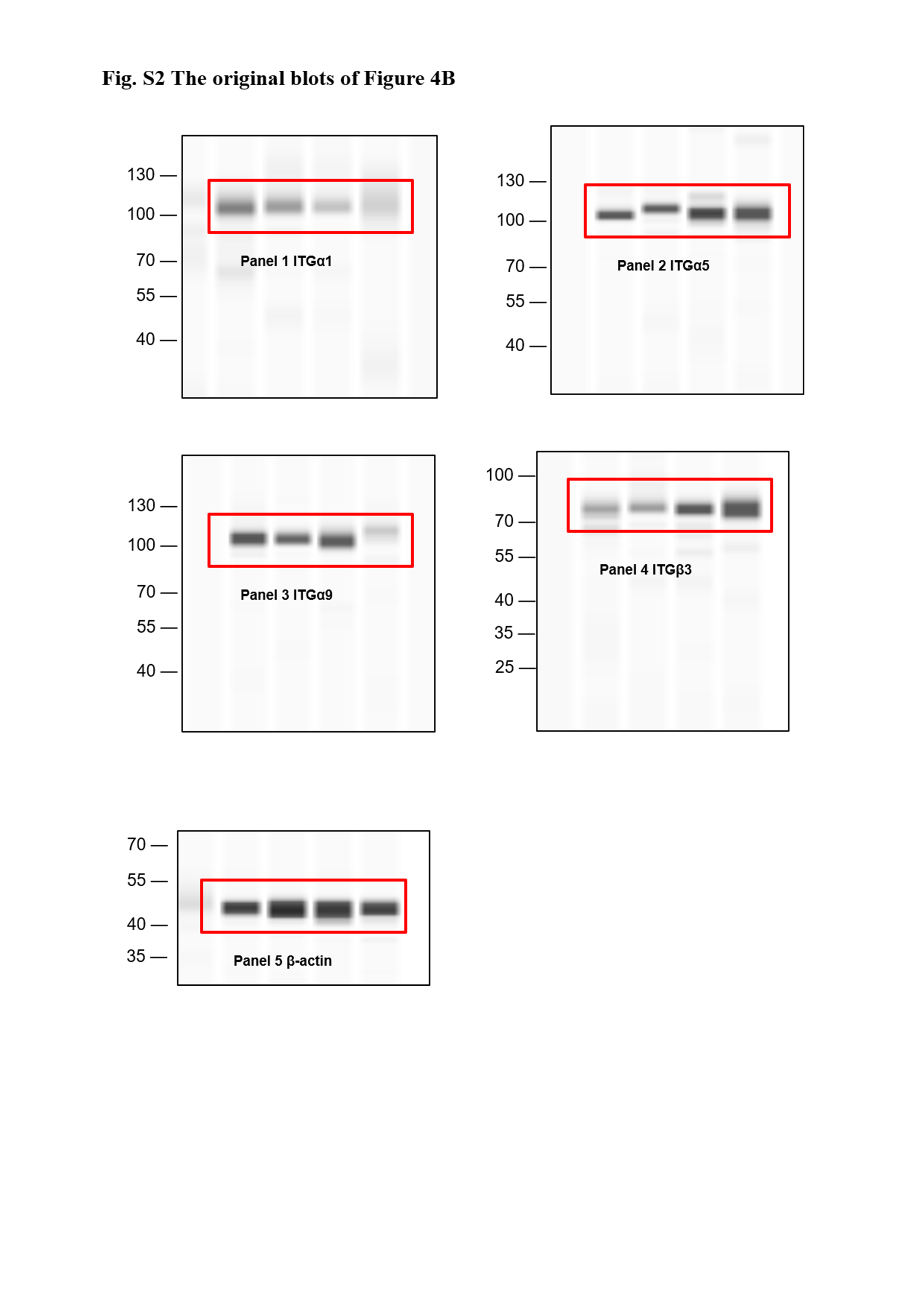

Supplement: Supplementary file 2 — Additional file 2: Fig. S2. The original blots of Fig. 4B. [file 12917_2022_3207_MOESM2_ESM.png]
